# PET-MRI nanoparticles imaging of blood–brain barrier damage and modulation after stroke reperfusion

**DOI:** 10.1093/braincomms/fcaa193

**Published:** 2020-11-11

**Authors:** Justine Debatisse, Omer Faruk Eker, Océane Wateau, Tae-Hee Cho, Marlène Wiart, David Ramonet, Nicolas Costes, Inés Mérida, Christelle Léon, Maya Dia, Mélanie Paillard, Joachim Confais, Fabien Rossetti, Jean-Baptiste Langlois, Thomas Troalen, Thibaut Iecker, Didier Le Bars, Sophie Lancelot, Baptiste Bouchier, Anne-Claire Lukasziewicz, Adrien Oudotte, Norbert Nighoghossian, Michel Ovize, Hugues Contamin, François Lux, Olivier Tillement, Emmanuelle Canet-Soulas

**Affiliations:** f1 Univ Lyon, CarMeN Laboratory, INSERM, INRA, INSA Lyon, Université Claude Bernard Lyon 1, 69000 Lyon, France; f2 Siemens-Healthcare SAS, Saint-Denis, France; f3 CREATIS, CNRS UMR-5220, INSERM U1206, Université Lyon 1, INSA Lyon Bât. Blaise Pascal, 7 Avenue Jean Capelle, Villeurbanne 69621, France; f4 Hospices Civils of Lyon, 69000 Lyon, France; f5 Cynbiose SAS, Marcy-L'Etoile, France; f6 CERMEP - Imagerie du Vivant, Lyon, France; f7 Laboratory of Experimental and Clinical Pharmacology, Faculty of Sciences, Lebanese University-Beirut, Lebanon; f8 Univ Lyon, Institut Lumière Matière, CNRS UMR5306, Université Claude Bernard Lyon 1, 69000 Lyon, France; f9 Institut Universitaire de France (IUF), France

**Keywords:** nanoparticles, stroke, blood–brain barrier, choroid plexus, ischaemia–reperfusion damage

## Abstract

In an acute ischaemic stroke, understanding the dynamics of blood–brain barrier injury is of particular importance for the prevention of symptomatic haemorrhagic transformation. However, the available techniques assessing blood–brain barrier permeability are not quantitative and are little used in the context of acute reperfusion therapy. Nanoparticles cross the healthy or impaired blood–brain barrier through combined passive and active processes. Imaging and quantifying their transfer rate could better characterize blood–brain barrier damage and refine the delivery of neuroprotective agents. We previously developed an original endovascular stroke model of acute ischaemic stroke treated by mechanical thrombectomy followed by positron emission tomography-magnetic resonance imaging. Cerebral capillary permeability was quantified for two molecule sizes: small clinical gadolinium Gd-DOTA (<1 nm) and AGuIX^®^ nanoparticles (∼5 nm) used for brain theranostics. On dynamic contrast-enhanced magnetic resonance imaging, the baseline transfer constant *K*_trans_ was 0.94 [0.48, 1.72] and 0.16 [0.08, 0.33] ×10^−3 ^min^−1^, respectively, in the normal brain parenchyma, consistent with their respective sizes, and 1.90 [1.23, 3.95] and 2.86 [1.39, 4.52] ×10^−3 ^min^−1^ in choroid plexus, confirming higher permeability than brain parenchyma. At early reperfusion, *K*_trans_ for both Gd-DOTA and AGuIX^®^ nanoparticles was significantly higher within the ischaemic area compared to the contralateral hemisphere; 2.23 [1.17, 4.13] and 0.82 [0.46, 1.87] ×10^−3 ^min^−1^ for Gd-DOTA and AGuIX^®^ nanoparticles, respectively. With AGuIX^®^ nanoparticles, *K*_trans_ also increased within the ischaemic growth areas, suggesting added value for AGuIX^®^. Finally, *K*_trans_ was significantly lower in both the lesion and the choroid plexus in a drug-treated group (ciclosporin A, *n* = 7) compared to placebo (*n* = 5). *K*_trans_ quantification with AGuIX^®^ nanoparticles can monitor early blood–brain barrier damage and treatment effect in ischaemic stroke after reperfusion.

## Teaser

Blood–brain barrier damage is the next target for neuroprotection after stroke ischaemia–reperfusion. Debatisse *et al.* used PET-MRI nanoparticles imaging for its precise quantification. Both brain infarct and choroid plexus permeabilities were significantly increased 2 h after recanalization. Finally, they were both decreased by a single ciclosporin A injection.

## Graphical Abstract

**Graphical Abstract:**
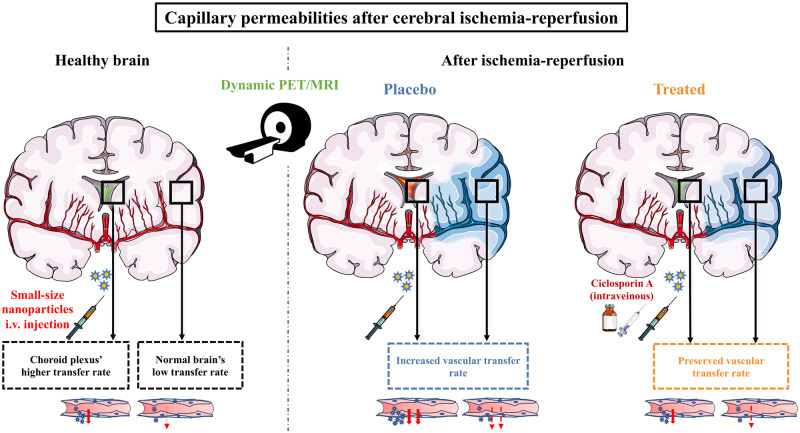

## Introduction

Blood–brain barrier (BBB) damage assessment is one of the major issues in acute ischaemic stroke. Current computed tomography (CT) and magnetic resonance imaging (MRI) methods in the context of acute stroke (Bai and Lyden, 2015) have been mainly applied during ischaemia to predict haemorrhagic transformation (Bivard *et al.*, 2020). Data after recanalization to evaluate ischaemia–reperfusion damage is still rare, and these methods are insufficient for accurate quantification of permeability to determine the degree of BBB damage. Gadolinium AGuIX^®^ nanoparticles (NPs), currently under clinical evaluation for theranostic application in radiation therapy, could meet this challenge (Sancey *et al.*, 2015). Firstly, their size is more suitable than the standard MRI or CT contrast agents to quantitatively evaluate a subtle change in BBB permeability. Secondly, depending on the imaging magnetic field, their higher r_1_ relaxivity per gadolinium molecule compared to commercial gadolinium chelates theoretically confers better MRI sensitivity. Finally, thanks to their chemical structure, fast and easy radiolabelling enables simultaneous detection of PET imaging, with its outcompeting nano- to pico-molar sensitivity. Moreover, they can be considered as future theranostic platforms for neuroprotective drug delivery.

The standard clinical MRI biomarker for BBB damage in stroke is known as the Hyperintense Acute Reperfusion Marker (HARM), corresponding to hyperintensity on post-gadolinium fluid-attenuated inversion recovery imaging (FLAIR); however, clinical data after recanalization of the occluded vessel are scarce (Luby *et al.*, 2019; Shi *et al.*, 2019). It is also difficult to evaluate the volume of the damage, as borders are not clearly delineated, and it does not provide quantitative measurements for analysis in peri-infarct areas. Early BBB leakage is thought to be greater in the ischaemic core, because of damage-associated molecular patterns and enzymes (MMP9) released from irreversible cell damage and subsequent inflammation activation (Villringer *et al.*, 2017; Shi *et al.*, 2019). The quantitative transfer constant *K*_trans_ is a sensitive measure of permeability in both normal and disease conditions to explore the BBB in the parenchyma and the specific barriers of the choroid plexus (Hubert *et al.*, 2019). However, few studies have evaluated its prognostic value in stroke (Russin *et al.*, 2018). Although BBB dysfunction is one of the major contributors to ischaemia–reperfusion injury, the dynamics of BBB abnormalities over time after recanalization are poorly documented. We therefore developed a methodological pipeline to evaluate *K*_trans_ in both pre- and post-stroke conditions in a non-human primate model. We first compared quantitative permeability between AGuIX^®^ NPs and small gadolinium chelates. We then hypothesized that *K*_trans_ measurement with AGuIX^®^ NPs (i) can serve as a biomarker of BBB damage after ischaemia–reperfusion and (ii) can monitor the modulation of permeability by drug treatment.

## Materials and methods

### Animals

All experiments were carried out in accordance with European Directive 2010/63/UE and the ARRIVE (Animal Research: Reporting in Vivo Experiments) guidelines, including critical examination for inclusion/exclusion criteria, randomization for treatment group allocation and blinded analysis of treatment at all time points. The study was approved by the Animal Welfare Body of Cynbiose and the Ethics Committee of VetAgro-Sup (C2EA-18) and CELYNE (C2EA-42), and authorized by the Ministry of Higher Education, Research and Innovation under registration numbers APAFIS#4702 and 8901. The animal facility of Cynbiose is fully accredited by the Association for Assessment and Accreditation of Laboratory Animal Care (AAALAC).

### Experimental model and study design

Mature male cynomolgus macaques (*Macaca fascicularis*) underwent middle cerebral artery occlusion followed by recanalization (*n* = 16). This thrombectomy-mimicking endovascular model in the non-human primate was previously described in detail (Debatisse *et al.*, 2020). In brief, an innovative endovascular procedure was set up to enable per-occlusion and post-recanalization PET-MRI imaging with minimal artefacts. During the endovascular procedure, animals were intubated and placed on a heating mattress, connected to the monitoring equipment (SCHILLER Maglife, Switzerland) via various sensors: heart and respiratory rate, ET-CO2, systolic, diastolic and mean arterial pressure and oxygen saturation to ensure a safe level of anaesthesia. Animals were imaged at five time points: (i) before any endovascular intervention (baseline for *n* = 5 animals), (ii) during occlusion, (iii) after recanalization, and (iv) 7 and (v) 30 days after stroke. Five minutes before recanalization of the occluded MCA, ciclosporin A (CsA, 2 mg/kg, Sandimmun^®^, Novartis, 50 mg ml^−1^, diluted in saline to 3.33 mg ml^−1^) or NaCl were injected via an intravenous (i.v.) catheter (*n* = 8 in each group). Animals were randomly assigned to the treatment group and investigators were blind to the treatment assignment during experimental procedures and data analysis. Exclusion criteria were lack of vascular occlusion or premature recanalization, characterized by the absence of core or ischaemic lesion on occlusion imaging. Animals were included in the study at the time of occlusion. Animals without follow-up or with insufficient imaging data quality were removed from the analysis. Standard clinical parameters including infarct size were evaluated (Debatisse *et al.*, 2020).

### AGuIX^®^ NPs and radiolabelling procedure

AGuIX^®^ NPs have been extensively characterized (Lux *et al.*, 2011; Mignot *et al.*, 2013) and are now under clinical evaluation for radiation therapy in various forms of cancer. Briefly, they are composed of a polysiloxane network with gadolinium chelates (GdSi_6.5_N_6_C_25_O_21_H_42_, 10H_2_O)_n_ conferring a high colloid stability and good monodispersity. Their weight in the order of 10 kDa and their ultra-small size with a hydrodynamic diameter of ∼5 nm enable fast renal elimination. Biodistribution and pharmacokinetics were previously assessed in both small and large animal models, with a mean blood half-life of 2.35 h in cynomolgus monkey (Kotb *et al.*, 2016*b*). Their MRI properties (∼10 gadolinium molecules per nanoparticle) ensure good T1 contrast at clinical imaging field level (*r*_1_/Gd = 11.4 s^−1^ mmol^−1^ at 60 MHz and 8.2 s^−1^ mmol^−1^ at 200 MHz) (Mignot *et al.*, 2013). They have a very low proportion of empty chelates, allowing fast radiolabelling for PET/SPECT imaging. For double-labelling AGuIX^®^ NPs production, [^68^Ga] was eluted in its chloride form as produced by a ^68^Ge/^68^Ga generator (GalliEo, IRE, Belgium) in 1.2 ml volume and placed in the radiolabeling reactor of a Neptis Mosaic^®^ mini Automatic synthesis unit. One millilitre AGuIX^®^ in acetate buffer (pH = 4.5) at 100 mg ml^−1^ was then added, and the mixture was allowed to react at 50°C for 10 min. Typically, 15 mCi (555 MBq) is recovered from a 20 mCi (740 MBq) elution after labelling.

Radiolabelling efficiency was checked by thin-layer chromatography with citrate buffer (0.25 M; pH = 5.2) using a radioactivity detector. Radiolabeled AGuIX^®^ NPs do not migrate within the layer. Purity is > 90%.

Gd-DOTA (Dotarem^®^, Guerbet, Aulnay-sous-bois, France) (0.5 kDa, r1 = 3.5 s^−1^ mmol^−1^) was used for comparison. Both contrast agents were injected at clinical dose for dynamic contrast-enhanced (DCE)-MRI measurements of 0.1 mmol Gd/kg (Villringer *et al.*, 2017), at 0.3 ml/s for AGuIX^®^ NPs and 3 ml s^−1^ for Gd-DOTA, followed by 10 ml saline flush at the same rates, using a power injector (MEDRAD^®^, Bayer, Switzerland).

### PET/MRI imaging protocol

**Figure 1:**
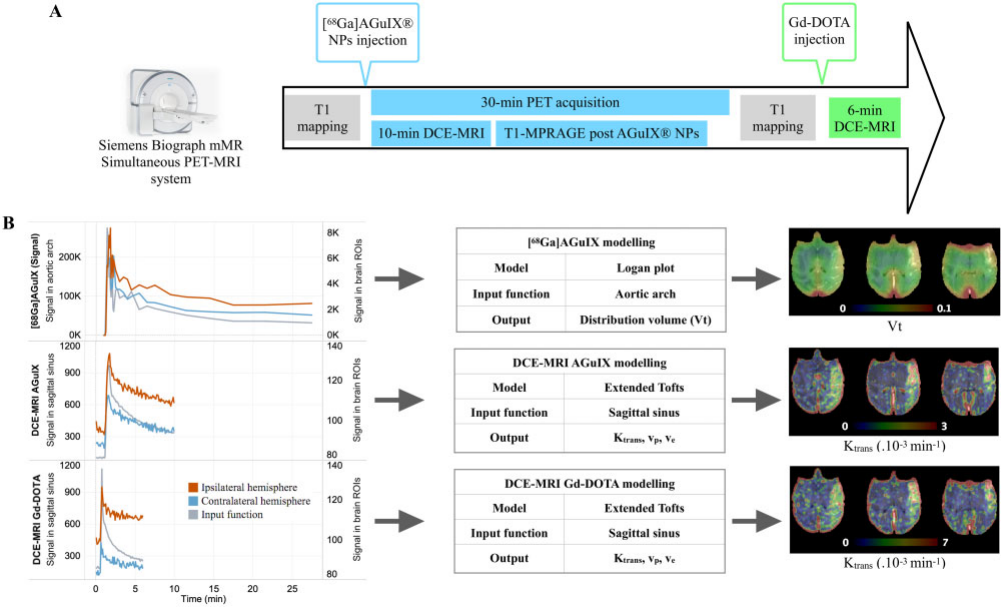
**Experimental set-up and study workflow.** Imaging data were acquired on a simultaneous PET-MRI system with T1 mapping, DCE-MRI acquisition with two contrast agents (AGuIX^®^ NPs and Gd-DOTA) (**A**). PET and MRI data were analysed (**B**) using Logan plot model for [^68^Ga]AGuIX^®^ NPs PET data to obtain parametric distribution volume (*V_t_*) images. AGuIX^®^ NPs and Gd-DOTA DCE-MRI data were analysed with Extended Tofts model to compute volume transfer constant (*K*_trans_), plasma volume (*V*_p_) and extravascular extracellular volume (*V_e_*) parametric maps. Input function was taken in the sagittal sinus. DCE-MRI = dynamic contrast-enhanced MRI; NPs = nanoparticles.

All animals were imaged on a fully integrated hybrid Biograph mMR PET-MRI 3 T scanner with 16-channel phased array head and two 110 mm loop-coils positioned on each side to optimize the signal-to-noise ratio (Siemens Healthcare, Erlangen, Germany). Prior to the PET-MRI session, a CT scan (Siemens Biograph mCT64, Siemens Healthcare, Erlangen, Germany) was obtained for each animal and used for PET attenuation correction. The MR imaging protocol included high-resolution pre- and post-contrast T1-weighted MPRAGE, T2 FLAIR, diffusion-weighted imaging (DWI), perfusion MRI (dynamic susceptibility contrast-MRI) and a DCE-MRI sequence was performed with each contrast agent to evaluate BBB status at baseline, post-recanalization, day +7 and day +30 (see above for contrast agent characteristics and injection protocol). Post-recanalization *K*_trans_ was measured ∼1 h after recanalization. Prior to each DCE-MRI and contrast agent administration, *T*_10_ was measured using four flip angles (5°, 10°, 15°, 25°) (Brookes *et al.*, 1999). MR acquisition parameters are detailed in Supplementary Table 1, and the experimental set-up is illustrated in Fig. 1A.

AGuIX^®^ NPs were dual-labelled with [^68^Ga] and dynamic PET data were acquired in 3D listmode over 30 min after [^68^Ga]AGuIX^®^ injection. PET data were reconstructed on a 256 × 256 matrix (voxel size: 0.7 × 0.7 × 2.03 mm^3^), 30 cm field-of-view, using point-spread function iterative reconstruction including random counts and dead time. Dynamic scans were attenuation-corrected using CT-based attenuation maps and were reconstructed in 31 frames: 16 × 5 s, 3 × 15 s, 3 × 30 s, 3 × 60s, 2 × 120s, 2 × 180 s, 1 × 240 s, 1 × 360 s.

Dynamic brain perfusion PET data were acquired over 6 min after bolus injection of radiotracer [^15^O]H_2_O (258 ± 8 MBq) followed by i.v. injection of 10 ml saline (injection rate, 3 ml s^−1^) using the power injector as previously described (Debatisse *et al.*, 2020).

### PET/MRI imaging analysis

High-resolution T1-weighted MPRAGE images acquired at each scanning session were used to obtain brain masks using the FMRIB Software Library (FSL; https://fsl.fmrib.ox.ac.uk/fsl) and were also used for transformation matrix calculation, using MincTools and in-house optimization tools (https://bic-mni.github.io/). Linear registration was used to co-register scanning sessions; non-linear wrapping was used to co-register imaging data on a common space using a *M. fascicularis* 3D template (Ballanger *et al.*, 2013).

Perfusion MRI was analysed on Olea Sphere clinical software, v3.0 (Olea Medical, La Ciotat, France), using manual arterial input function in the unaffected MCA and oSVD deconvolution parameters to compute rCBV maps.

Image-derived input functions (IDIF) were extracted from the dynamic [^68^Ga]AGuIX^®^ NPs PET and [^15^O]H_2_O perfusion PET data using a 6 mm-diameter spherical volume of interest (VOI) located in the aortic arch (Amide v1.0.5). For [^68^Ga]AGuIX^®^ NPs PET, a Logan plot was used to quantify the volume of distribution in brain tissue (*V_t_*) and to derive parametric quantitative *V_t_* maps (http://www.turkupetcentre.net/petanalysis/) (Fig. 1B). Distribution volume was defined as the ratio of tracer concentrations in tissue and in plasma at equilibrium and was expressed as mL plasma per cm^−3^ tissue. For [^15^O]H_2_O PET, absolute PET-CBF parametric maps were computed using a one-tissue-compartment and ischaemic penumbra was defined as brain tissue with CBF < 0.2 ml g^−1^ min^−1^ as previously validated (Debatisse *et al.*, 2020).

DCE-MRI data with both contrast agents (AGuIX^®^ NPs and Gd-DOTA) were analysed with the Olea Sphere software, v3.0 SP10 (Olea Medical, La Ciotat, France), using the Extended Tofts model (Tofts *et al.*, 1999). *T*_10_ maps were used to convert the MR signal into gadolinium concentration, enabling the quantitative measurement of *K*_trans_. Relaxivity *r*_1_ was fixed at 3.5 s^−1^ mmol^−1^ for Gd-DOTA and 8.9 s^−1^ mmol^−1^ for AGuIX^®^ NPs. The input function was manually defined from the sagittal sinus. Several studies showed that selecting a venous input function instead or the usual arterial input function could be a useful compromise if no large vessel is in the field-of-view (Sourbron *et al.*, 2009; Heye *et al.*, 2016). Parametric maps of the volume transfer constant *K*_trans_, expressed in ×10^−3 ^min^−1^, were obtained for both contrast agents at all imaging time-points (Fig. 1B). For further comparison, *K*_trans_ was analysed for both agents using the Olea sphere, with or without *T*_1_ adjustment from *T*_10_ mapping (absolute versus relative *K*_trans_, respectively).

Lesion expansion within the ischaemic area was evaluated by standard DWI b1000, apparent diffusion coefficient (ADC) maps computed from DWI imaging and T2 FLAIR. MRI and lesion volumes were segmented by an experienced neurologist (THC). Acute infarct (or ischaemic core) was defined as the lesion volume segmented from per-occlusion DWI imaging, infarct at Day 7 as lesion volume segmented on Day 7 T2 FLAIR, and infarct at Day 30 as lesion volume segmented on Day 30 T2 FLAIR. During occlusion, the ischaemic area was defined by ^15^O water PET, with a PET cerebral blood flow (CBF) threshold of 0.2 ml g^−1^min^−1^, using validated procedures (Debatisse *et al.*, 2020). The penumbra (i.e. ischaemic tissue not yet in the core) and two progressive tissue compartments [i.e. asymptomatic infarcted tissue (progression area) and symptomatic salvaged tissue (regression area)] were defined as previously described (Supplementary Fig. 1A) (Debatisse *et al.*, 2020). Progression area was defined as voxels neither hypoperfused nor in the DWI core lesion but inside the Day-7 FLAIR lesion. The regression area was defined as voxels either hypoperfused or in the DWI core lesion per-occlusion but outside the Day-7 FLAIR lesion.

### Comparison of permeability with AGuIX^®^ NPs versus Gd-DOTA

Baseline imaging and the placebo group were used to characterize permeability with AGuIX^®^ NPs in comparison with the clinical gadolinium chelate (*n* = 5 animals). Measurements were made in both brain parenchyma and choroid plexus to explore a wide range of physiological values. In brain parenchyma, small 4-mm circular regions of interest (ROI) were manually traced in both hemispheres. Permeability in the choroid plexus was defined as the median value over the whole of the lateral ventricles. At post-intervention time-points, ROIs were placed in the acute infarct (lesional and peri-lesional areas) and their respective contralateral mirrors (Supplementary Fig. 1B). To check for the influence of blood flow on permeability parameters, PET-CBF was measured at each time-point in the same regions. To evaluate how T1 changes following ischaemia–reperfusion impacted permeability with AGuIX, post-recanalization *K*_trans_ values with (absolute *K*_trans_) and without intrinsic T1 values (relative *K*_trans_) were compared. Relative *K*_trans_ used pharmacokinetic modelling after signal intensity correction for mean pre-contrast intensity, whereas absolute *K*_trans_ used pharmacokinetic modelling after conversion to local contrast agent concentration, calculated from pre-injection T1 mapping. ROIs with T1 values >5 s and negative *K*_trans_ values were considered artefactual and were removed.

### Evaluation of AGuIX^®^ NPs *K*_trans_ as a biomarker of ischaemia–reperfusion injury

Median ADC and *K*_trans_ values were extracted from parametric maps for the acute lesion during occlusion (DWI), the established infarct at Day 7 (T2 FLAIR), the reversible ischaemic region (regression area) and the finally infarcted previously asymptomatic (progression) region, and were normalized by their contralateral counterparts, expressed as ratios.

### Blood and tissue analysis

MMP9, an established early plasma marker of BBB damage (Jha *et al.*, 2014), was measured 10 min after recanalization in blood samples using an ELISA test. Tubes were centrifugated for 5 min at 500×g and plasma was separated and distributed in small aliquots and stored at −80°C for the MMP9 determination. MMP9 plasma level was determined by ELISA using the ab246539 kit from Abcam following the manufacturer's protocol.

### Statistical analysis

Values are presented as median [first quartile: Q1, third quartile: Q3].

One-way non-parametric repeated measures analysis of variance (ANOVA) (Friedman test) was used to analyse the longitudinal progression of imaging parameters, followed by *post hoc* Dunn’s test for multiple comparison.

Two-way repeated measures analysis of variance (ANOVA) was used to longitudinally compare lesion size in the two treatment groups, followed by *post hoc* Tukey or Bonferroni test for multiple comparison. Data were Log transformed when normality was not respected.

A non-parametric paired Wilcoxon test was used to compare ipsi- versus contra-lateral hemisphere ROI values and to compared imaging parameters between imaging sessions.

Non-parametric Mann–Whitney test was used to compare imaging parameters between treatment groups. Statistical significance was corrected for multiple comparison.

All statistical analyses used GraphPad Prism software, version 6 (GraphPad Software, La Jolla, CA, USA).

### Data availability

All data generated or analysed during this study are included in this published article and its Supplementary material.

## Results

### Animals

All the 16 animals were included. Supplementary Table 2 provides the monitoring parameters enabling a stable median occlusion duration of 112 min [Q1 = 109; Q3 = 114 min]. There was one death in each group within the first 24 h: one from haemorrhage after severe hypoperfusion (<10 ml min^−1^100 g^−1^ in the core) due to poor collaterals and a fast-growing lesion during ischaemia (Supplementary Fig. 2), and the second from recanalization failure due to a defective endovascular device. The remaining 14 animals, (*n* = 7 in each treatment group) completed each imaging time-point. Two control animals did not have good quality DWI and DCE images due to movement during acquisition and were excluded (Supplementary Fig. 2B).

### Characterization of *K*_trans_ and *V_t_* measurements with AGuIX^®^ NPs

Prior conversion-to-concentration curve, representative PET activity and MRI signal enhancement curves are presented in Fig. 1, for blood and for normal and ischaemic brain parenchyma. The signal range confirmed higher sensitivity for PET (Fig. 1B). The brain tissue curve derived from post-recanalization [^68^Ga]AGuIX^®^ NPs PET, DCE-MRI with AGuIX^®^ NPs and DCE-MRI with Gd-DOTA (Fig. 1B) showed similar blood-to-tissue signal dynamics, enabling kinetic modelling. The initial higher signal intensity in the ischaemic hemisphere (ROIs located in high permeability areas on post-gadolinium T1 image) did not alter the dynamic range of the signal enhancement curves. With small Gd-DOTA chelates, the curve reached a plateau and steady-state signal earlier (Fig. 1B) than with AGuIX^®^ NPs, typical of small versus larger molecule vascular kinetics.

On baseline imaging before any endovascular intervention, we measured median basal permeability values in both hemispheres (*n* = 5 animals) and in choroid plexus (Table 1). *K*_trans_ values in the ipsilateral hemisphere (0.16 [0.08, 0.33] and 0.94 [0.48, 1.72] × 10^−3 ^min^−1^ with AGuIX^®^ NPs and Gd-DOTA, respectively) were consistent with very low baseline permeability for both agents in cerebral tissue (*K*_trans_ < 1 × 10^−3 ^min^−1^) but were related to their respective molecular weights (four times higher for the smaller molecular weight agent). Choroid plexus is known to display higher permeability than brain parenchyma, but there are at present no established clinical values, whether in healthy or diseased tissue (Hubert *et al.*, 2019). In the more permeable choroid plexus, *K*_trans_ was higher (2.86 [1.39, 4.52] and 1.90 [1.23, 3.95] × 10^−3 ^min^−1^ with AGuIX^®^ NPs and Gd-DOTA respectively in the ipsilateral ventricle) and similar for both agents (Table 1). *V_t_* values, however, did not show such a difference. Spatial resolution was lower on Gallium-PET (Sanchez-Crespo, 2013) than MRI, and the partial volume effect in the small structures of the choroid plexus may attenuate distribution volume (*V_t_*) values in this region (Table 1). PET cerebral blood flow (CBF) and MRI relative blood volume (rBV) values measured in the same regions are shown in Table 1. There was no relationship between permeability values and perfusion/blood volume values, confirming the relevance of the chosen kinetic models (extended Tofts for MRI and distribution volume for PET).

**Table 1:** Baseline permeability (PET AGuIX^®^ NPs *V_t_*, MRI AGuIX^®^ NPs *K*_trans_, MRI Gd-DOTA *K*_trans_), blood flow (PET-CBF) and blood volume (MRI-rBV) values in the ipsilateral and corresponding contralateral hemisphere and in choroid plexus

	Ipsilateral hemisphere	Contralateral hemisphere	Ipsilateral lateral ventricle (choroid plexus)	Contralateral lateral ventricle (choroid plexus)
**AGuIX^®^ NPs** ***V_t_*** **(ml cm^−3^)**	0.10 [0.05, 0.12]	0.09 [0.05, 0.11]	0.08 [0.05, 0.10]	0.06 [0.05, 0.11]
**AGuIX^®^ NPs** ***K*_trans_** **(**×**10^−3 ^min^−1^)**	0.16 [0.08, 0.33]^a^	0.21 [0.12, 0.31]^a^	2.86 [1.39, 4.52]	2.78 [1.94, 4.15]
**Gd-DOTA** ***K*_trans_** **(**×**10^−3 ^min^−1^)**	0.94 [0.48, 1.72]^a^	0.80 [0.47, 1.72]^a^	1.90 [1.23, 3.95]	2.52 [1.04, 3.43]
**PET-CBF (ml g** ^−1^ **min**^−1^**)**	0.54 [0.47, 0.61]	0.53 [0.47, 0.61]	0.46 [0.45, 0.48]	0.46 [0.45, 0.49]
**MRI-rBV (%)**	3.01 [2.32, 4.09]	2.56 [1.82, 4.01]	4.63 [4.13, 6.98]	4.37 [4.05, 7.33]

Values are expressed as median [Q1: first quartile, Q3: third quartile]. In each hemisphere, *n* = 98 ROIs. *N* = 5 animals in both treated and non-treated groups.

Non-parametric paired Wilcoxon test compared AGuIX^®^  *K*_trans_ and Gd-DOTA *K*_trans_ (^a^). *P*-values <0.0125 were considered significant and corrected for multiple comparisons.

NPs = nanoparticles; PET-CBF = positron emission tomography cerebral blood flow; rBV = relative blood volume; *V_t_* = distribution volume.

**Figure 2:**
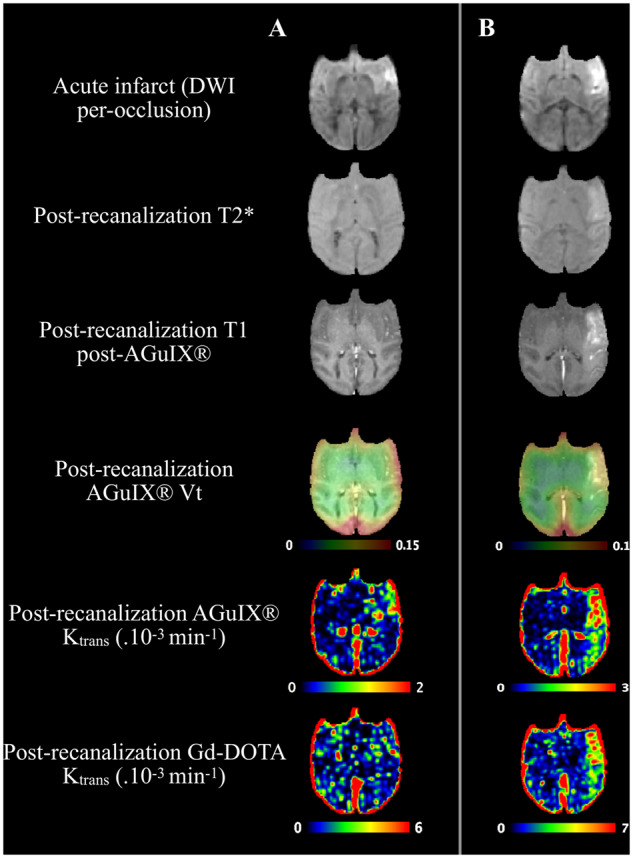
**Representative illustrations of abnormal BBB permeabilities.** Illustrations of diffusion-weighted scan (acute infarct), post-contrast T1w scan, T2* and BBB permeability maps (from fourth to sixth row, PET AGuIX^®^ NPs *V_t_* overlaid on T1 MRI, MRI AGuIX^®^ NPs *K*_trans_, MRI Gd-DOTA *K*_trans_, respectively) in two animals with focal and limited BBB leakage (**A**) and extended parenchymal and subarachnoid BBB leakage (**B**). BBB = blood–brain barrier; NPs = nanoparticles.

After ischaemia–reperfusion, parametric maps obtained with AGuIX^®^ NPs and Gd-DOTA both identified regions in the ipsilateral hemisphere with abnormal BBB permeability (Fig. 2), from small BBB leakage (Supplementary Fig. 2A) to extensive subarachnoid and parenchymal BBB leakage (Fig. 2B) and haemorrhage (Supplementary Fig. 2). Compared to standard post-contrast T1-weighted images, quantitative AGuIX^®^ NPs *K*_trans_ maps allowed delineation of small leakages (Fig. 2A) with better contrast than in the contralateral hemisphere. PET-derived *V_t_* also allowed location of large BBB leakages (Fig. 2B), but was less accurate for smaller ones (Fig. 2A), due to the lower spatial resolution of ^68^Gallium PET imaging and partial volume effects.

To characterize the range of permeability changes over time, analysis was first performed in ROIs. As BBB leakage after ischaemia–reperfusion was not only observed in the lesion area but also in the surrounding perilesional area (Fig. 2 and Supplementary Fig. 2), ROIs were placed in both areas in the ipsilateral hemisphere and in symmetrical contralateral regions (Supplementary Fig. 1B), carefully checking to avoid large vessels. The influence of higher T1 values in the ischaemic regions was assessed on absolute and relative *K*_trans_ maps. Correlations were excellent between the two parameters in the pathological hemisphere (for AGuIX^®^ NPs, Supplementary Fig. 3A, *r*^2^ = 0.74, regression slope 1.4; and for Gd-DOTA, Supplementary Fig. 3B, *r*^2^ = 0.67, regression slope 1.6). Both AGuIX^®^ NPs and Gd-DOTA absolute and relative *K*_trans_ maps showed very similar location and intensity of BBB leakage areas despite local variations in pre-contrast intrinsic T1 value, as illustrated in Supplementary Fig. 3C. Perilesion ROIs showed ranges of values similar to lesion ROIs (Supplementary Fig. 3A) and were therefore pooled for the rest of the ROI analysis.

Progression of permeability and clinical imaging diffusion and perfusion parameters are shown in Table 2. As expected, ADC values significantly decreased during ischaemia and post-recanalization compared to contralateral mirror values and increased at day + 7 after ischaemia–reperfusion (Supplementary Fig. 4A). A similar pattern was observed in pre-contrast T1-values, with a decrease at the post-recanalization imaging session and an increase at day + 7 in the hemisphere ipsilateral to the lesion (Supplementary Fig. 4B). Perfusion data (CBF derived from [^15^O]H_2_O-PET analysis) showed significantly lower CBF in ipsilateral (0.28 [0.23, 45] ml g^−1^ min^−1^) than contralateral ROIs (0.42 [0.31, 0.52] ml g^−1^ min^−1^) after recanalization (Table 2). Median CBF values after recanalization were above the ischaemic threshold used to characterize the ischaemic area (i.e. 0.2 ml g^−1^ min^−1^) (Table 2). Post-recanalization AGuIX^®^ NPs *K*_trans_, AGuIX^®^ NPs *V_t_* and Gd-DOTA *K*_trans_ were significantly increased in the ipsilateral hemisphere compared to symmetrical contralateral regions (Table 2). In choroid plexus, a similar pattern was obtained at the post-recanalization and Day 7 imaging sessions in the placebo group (Table 3), with a median post-recanalization AGuIX^®^ NPs *K*_trans_ of 3.3 [3.1, 6.7] and Gd-DOTA *K*_trans_ of 2.6 [2.4, 3.9] in the ipsilateral lateral ventricles (Table 3). There was no significant difference with the contralateral choroid plexus value. Of note, *K*_trans_ in the haemorrhagic core was even higher, 3-fold compared to the choroid plexus (Supplementary Fig. 2). For gallium, spatial resolution was too low and partial volume effects affected *V_t_* measurements in the choroid plexus.

**Table 2:** Post-recanalization and Day +7 permeability (PET distribution volume AGuIX^®^ NPs *V_t_*, MRI AGuIX^®^ NPs *K*_trans_, MRI Gd-DOTA *K*_trans_), blood flow (PET-CBF) and blood volume (MRI-rBV) values in the ipsilateral and corresponding contralateral hemisphere

	Post-recanalization ipsilateral	Post-recanalization contralateral	Day +7 ipsilateral	Day +7 contralateral
**AGuIX^®^ NPs *V_t_* (ml cm^−3^)**	0.08 [0.05, 0.11]^a,b^	0.07 [0.05, 0.09]^a,b^	0.06 [0.05, 0.07]^b^	0.06 [0.05, 0.07]^b^
**AGuIX^®^ NPs** ***K*_trans_** **(**×**10^−3 ^min^−1^)**	0.82 [0.46, 1.87]^a,b^	0.36 [0.14, 0.90]^a^	0.39 [0.18, 0.75]^b^	0.44 [0.20, 0.85]
**Gd-DOTA** ***K*_trans_** **(**×**10^−3 ^min^−1^)**	2.23 [1.17, 4.13]^a,b^	1.13 [0.58, 1.95]^a,b^	0.95 [0.37, 1.84]^b^	0.85 [0.31, 1.27]^b^
**PET-CBF (ml g** ^−1^ **min**^−1^**)**	0.28 [0.23, 0.45]^a,b^	0.42 [0.31, 0.52]^a,b^	0.27 [0.21, 0.35]^b^	0.29 [0.23, 0.34]^b^
**MRI-rBV (%)**	2.34 [1.76, 3.97]^a,b^	2.11 [1.59, 2.85]^a,b^	3.06 [1.91, 4.91]^b^	2.73 [1.82, 4.89]^b^

Values are expressed as median [Q1: first quartile, Q3: third quartile]. *N* = 5 animals of the non-treated group, *n* = 96 ROIs. Non-parametric paired Wilcoxon test compared brain side (ipsilateral versus contralateral ^a^) and scanning session (post-recanalization versus day +7 ^b^).

*P*-values <0.0125 were considered significant and corrected for multiple comparisons.

NPs = nanoparticles; PET-CBF = positron emission tomography cerebral blood flow; rBV = relative blood volume; *V_t_* = distribution volume.

**Table 3:** Post-recanalization and day +7 permeability (PET distribution volume AGuIX^®^ NPs Vt, MRI AGuIX^®^ NPs *K*_trans_, Gd-DOTA *K*_trans_), blood flow (PET-CBF) and blood volume (MRI-rBV) in the ipsilateral and contralateral lateral ventricles (choroid plexus)

	Post-recanalization ipsilateral ventricle	Post-recanalization contralateral ventricle	Day +7 ipsilateral ventricle	Day +7 contralateral ventricle
**AGuIX^®^ NPs** ***V_t_*** **(ml cm^−3^)**	0.06 [0.05, 0.09]	0.06 [0.05, 0.08]	0.07 [0.06, 0.07]	0.07 [0.05, 0.07]
**AGuIX^®^ NPs** ***K*_trans_** **(**×**10^−3 ^min^−1^)**	3.3 [3.1, 6.7]	4.1 [3.4, 4.6]	7.8 [4.7, 20.2]	4.1 [3.7, 10.2]
**Gd-DOTA** ***K*_trans_** **(**×**10^−3 ^min^−1^)**	2.6 [2.4, 3.9]	2.7 [2.6, 3.8]	3.5 [1.2, 21.8]	3.3 [0.7, 8.4]
**PET-CBF (ml g** ^−1^ **min**^−1^**)**	0.39 [0.26, 0.41]	0.40 [0.28, 0.42]	0.26 [0.20, 0.27]	0.26 [0.20, 0.27]
**MRI-rBV (%)**	3.61 [3.42, 6.39]	3.83 [2.69, 5.51]	4.96 [4.32, 6.88]	4.50 [3.15, 5.21]

Values are expressed as median [Q1: first quartile, Q3: third quartile]. *N* = 5 animals of the non-treated group.

NPs = nanoparticles; PET-CBF = positron emission tomography cerebral blood flow; rBV = relative blood volume; *V_t_* = distribution volume.

Permeability measurements did not correlate with perfusion values at baseline or at reperfusion, confirming the relevance of the chosen kinetic models (extended Tofts for MRI and distribution volume for PET). AGuIX^*^®^*^ NPs *K*_trans_ was successfully validated and is used below for follow-up of pathology and treatment effect, normalized to contralateral values.

### Evaluation of AGuIX^®^ NPs *K*_trans_ as a biomarker of ischaemia–reperfusion damage

**Figure 3:**
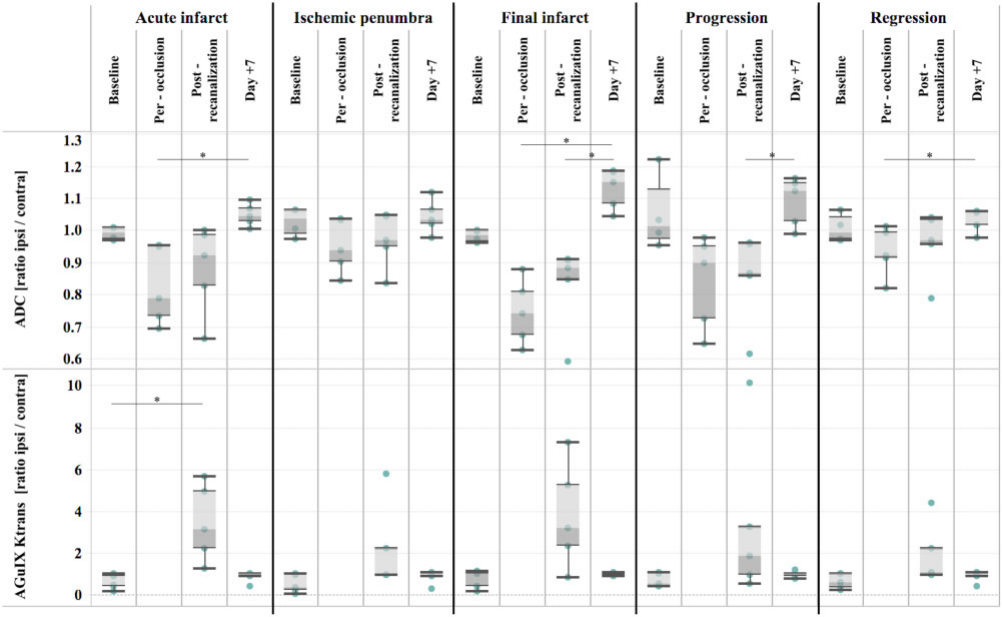
**Extension of oedema (decreased ADC) and BBB damage (increased permeability) to all compartments, including apparently remote regions at early reperfusion.** Boxplot of baseline, per-occlusion, post-recanalization, Day+7 ADC, AGuIX^®^ NPs *K*_trans_ in the five compartments: acute infarct, ischaemic penumbra, final infarct, progression and regression areas (*n* = 5 animals). Data are normalized by their contralateral value and expressed as ipsilateral/contralateral ratio. *P-value < 0.05 one-way repeated measures non-parametric ANOVA (Friedman test) followed by Dunn's multiple comparisons test. ADC = apparent diffusion coefficient; BBB = blood–brain barrier; NPs = nanoparticles.

The clinical HARM sign of BBB leakage (i.e. positive enhancement on post-contrast FLAIR) was present after recanalization in all five subjects of the placebo group and no longer present at Day 7. Both oedema (decreased ADC) and permeability (increased AGuIX^*^®^*^ NPs *K*_trans_) were then evaluated longitudinally in the following regions: the acute lesion during occlusion (DWI), the ischaemic penumbra, the established infarct at Day 7 (T2 FLAIR), the reversible ischaemic or reversible DWI-positive region (regression) and the asymptomatic but finally infarcted region (progression) (Fig. 3). For ADC, there was a significant effect of imaging session in all regions (acute infarct: *P* < 0.01, *F* = 11.64; final infarct: *P* < 0.01, *F* = 14.04; ischaemic penumbra: *P* < 0.05, *F* = 8.28; progression: *P* < 0.01, *F* = 12.12; regression: *P* < 0.05, *F* = 9.72). Dunn test for multiple comparison showed significant differences between per-occlusion and Day 7 ADC values in acute infarct (*P* < 0.01), final infarct (*P* < 0.01) and regression (*P* < 0.05) and between post-recanalization and Day 7 ADC values in final infarct (*P* < 0.05) and progression (*P* < 0.05). For AGuIX^®^ NPs *K*_trans_, Friedman test showed significant results in acute infarct only (*P* < 0.05, *F* = 7.6) and Dunn test for multiple comparisons showed significant differences between baseline and post-recanalization *K*_trans_ values (*P* < 0.05) (Fig. 3).

### Permeability to AGuIX^®^ NPs is lowered by CsA treatment

Lesion volume progression in the CsA-treated and placebo groups can be seen in Table 4. In the placebo group, median acute infarct core volume during occlusion (DWI) was 2.6 [2, 4] ml, final infarct at Day 7 (T2 FLAIR) 2.7 [0.5, 2.8] ml, ischaemic penumbra 5.2 [3.6, 12.2] ml, regression zone 2 [1, 11.2] ml, and progression zone 0.5 [0.1, 1.1] ml. Two-way repeated measures ANOVA did not show an effect of treatment on lesion size but *post hoc* Tukey analysis showed significant differences between acute infarct and infarct at Day 30 in both groups (Table 4). The difference between CsA and placebo groups did not reach significance for penumbra, regression or progression region volumes (Table 4).

**Table 4:** Volumes of acute infarct (DWI per-occlusion lesion), infarct Day 7 (FLAIR Day 7 lesion), infarct Day 30 (FLAIR Day 30 lesion), ischaemic penumbra, progression and regression in placebo and CsA treatment groups

	Median [Q1, Q3] volume in ml	
	Placebo (*n* = 5)	CsA (*n* = 7)
**Acute infarct (DWI per-occlusion lesion)**	2.6 [2.0, 4.0]^a^	2.3 [0.8, 4.2]^a^
**Infarct Day 7 (FLAIR Day 7 lesion)**	2.7 [0.5, 2.8]	0.6 [0.3, 2.6]
**Infarct Day 30 (FLAIR Day 30 lesion)**	0.1 [0.1, 0.3]^a^	0.05 [0.01, 0.3]^a^
**Ischaemic** **penumbra**	5.2 [3.6, 12.2]	2.9 [1.7, 5.5]^b^
**Progression**	0.5 [0.1, 1.1]	0.2 [0.2, 1.2]^b^
**Regression**	2.0 [1.0, 11.2]	0.9 [0.4, 1.8]^b^

Values are expressed as median [Q1: first quartile, Q3: third quartile]. Two-way repeated measures ANOVA compared longitudinal progression of lesion and treatment effect, followed by Dunn’s test for multiple comparisons.

a *P*-value < 0.05: significant differences between acute infarct and infarct Day 30.

b *n* = 6 (one animal had PET-CBF values above the ischaemic threshold of 0.2 ml min^−1^ g^−1^).

CsA = ciclosporin A; DWI = diffusion-weighted imaging; FLAIR = fluid-attenuated inversion recovery.

**Figure 4:**
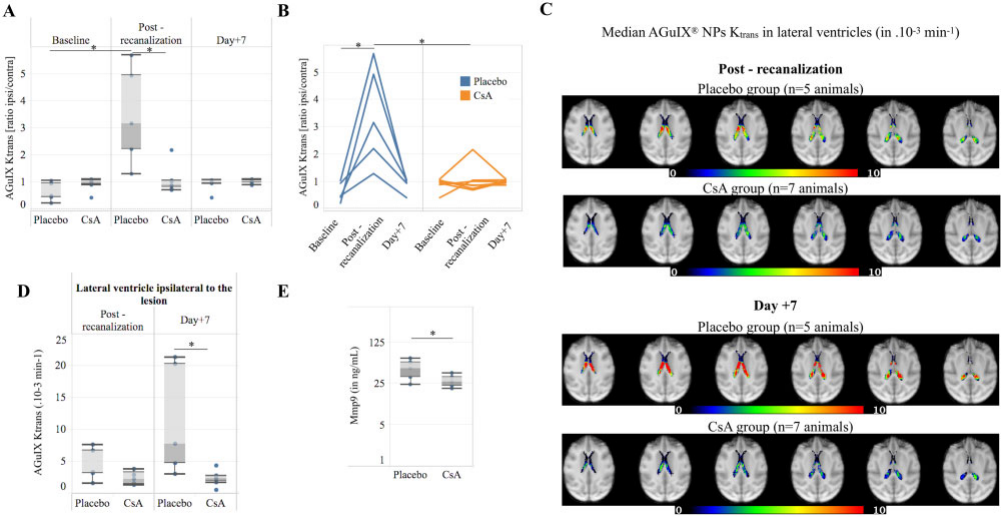
**Permeability to AGuIX^®^ NPs is lowered by CsA treatment in brain parenchyma and choroid plexus.** Longitudinal progression of normalized BBB permeability (AGuIX^®^ NPs *K*_trans_) in the acute infarct in the two treatment groups represented as boxplots (**A**) or individual values (**B**). Median AGuIX^®^ NPs *K*_trans_ at post-recanalization and Day +7 lateral ventricles in Placebo (*n* = 5 animals) and CsA (*n* = 7 animals) groups in choroid plexus (**C**). Although not significantly, the choroid plexus in the ipsilateral hemisphere showed decreased *K*_trans_ in the CsA-treated group post-recanalization (*n* = 7 animals) compare to the placebo group (*n* = 5 animals), which persisted and became significantly lower at Day 7 in the group treated with CsA (**D**). MMP9 levels at the post-recanalization was also significantly lower in the treated group (*n* = 5 in each group) (**E**). (**A**) * *P* < 0.05 two-way repeated measures ANOVA followed by *post hoc* Bonferroni test for multiple comparisons. (**D, E**) * *P* < 0.05 non-parametric Mann–Whitney test. BBB = blood–brain barrier; CsA = ciclosporin A; NPs = nanoparticles.

Similar to the placebo group, the clinical HARM sign of BBB leakage was present after recanalization in all seven of the CsA group. Two-way repeated measures ANOVA showed a significant effect of treatment on AGuIX^®^ NPs *K*_trans_ in acute infarct [*P* < 0.01, *F*(2,20) = 13.69] and *post hoc* Bonferroni analysis showed significant differences between post-recanalization AGuIX^®^ NPs *K*_trans_ values in both treatment groups (*P* < 0.01) (Fig. 4A and B for individual progressions). The same significant effect of treatment was found for final infarct AGuIX^®^ NPs *K*_trans_ [*P* < 0.05, *F*(2,20) = 7.7] and *post hoc* Bonferroni analysis showed significant differences between post-recanalization AGuIX^®^ NPs *K*_trans_ values in both treatment groups (*P* < 0.01) (Supplementary Fig. 5A).

In both groups, median AGuIX^®^ NPs *K*_trans_ maps at post-recanalization and at Day +7 exhibited similar well-delineated focal higher-permeability areas in the lateral ventricles corresponding to choroid plexus with no visible leakage in the ventricles (Fig. 4C). The choroid plexus in the ipsilateral hemisphere showed a lower post-recanalization *K*_trans_ (AGuIX^®^ NPs) in the CsA-treated group compared to the placebo. On Day 7, *K*_trans_ was significantly lower in the treated group compared to the increased *K*_trans_ (compared to post-recanalization) in the placebo group (Fig. 4D). The same result was observed in the contralateral lateral ventricle (Supplementary Fig. 5E). MMP9 levels at post-recanalization were also significantly lower in the treated group (Fig. 4E).

## Discussion

We demonstrated for the first time that MRI NPs enabled robust *in vivo* quantitative measurement of brain barrier permeability including BBB and the choroid plexus barrier in both normal and pathological conditions. We were able to characterize the progression of subtle BBB abnormalities after acute ischaemic stroke in the initial lesion core, penumbra and evolutive regions (progression or regression), thereby providing a basis to understand the dynamics of one of the key components of ischaemia–reperfusion injury. Finally, we found that ciclosporin A had a modulatory effect on the BBB and at the choroid plexus by reversing *K*_trans_ increase after recanalization. More work is needed to elucidate the exact mechanisms of this effect.

### Interest of *K*_trans_ in characterizing BBB and choroid plexus permeability to NPs

Evaluating dynamic permeability in the brain is still a challenging task, as not many methods enable non-invasive *in vivo* measurement in the whole brain. In clinical stroke applications, DCE-MRI with small gadolinium chelates is the only recognized technique [5,16]. Recent preclinical studies established the mechanisms of water and small solute passage and their consequences in acute stroke (Knowland *et al.*, 2014; Mestre *et al.*, 2020). However, studies were mainly performed in permanent ischaemia and much less in ischaemia–reperfusion. In preclinical stroke models, BBB leakage is often evaluated at a single time point using albumin-bound Evans Blue dye or postmortem staining by immunoglobulin G, which are comparatively large molecules. In the limited number of clinical papers with evaluation of post-thrombectomy BBB leakage (Renú *et al.*, 2017), HARM sign after gadolinium chelate was mainly evaluated in the subarachnoid space. To our knowledge, there is very little published DCE data in stroke patients (Simpkins *et al.*, 2016; Merali *et al.*, 2017; Villringer *et al.*, 2017). After thrombolysis, Villringer *et al.* (2017) showed a 2-fold increase in *K*_trans_ in the ischaemic core using a gadolinium chelate, compared to the contralateral value (0.6 and 0.3 × 10^−3 ^min^−1^, respectively), and there was no association between higher *K*_trans_ and HARM sign. The present study also found no association with HARM sign (present in all cases) and a 2.3-fold ratio with AGuIX NPs (0.8 and 0.4 × 10^−3 ^min^−1^, respectively) and 2-fold ratio with Gd-DOTA (2.2 and 1.1 ×10^−3 ^min^−1^, respectively) (Table 2). Interestingly, with both agents, the contralateral *K*_trans_ after recanalization was also almost twice the baseline value (Table 1).

An important finding of the present study was the confirmation of *K*_trans_ as a robust parameter assessing permeability. Despite greater sensitivity, PET *V_t_* with Gallium-labelled NPs was not able to evaluate subtle permeability increase or different values in small regions such as choroid plexus, due to its limited spatial resolution of 7 mm (defined by the full width at half maximum in PET) (Sanchez-Crespo, 2013). For permeability measurements, assumptions are needed to run the proper model, and recommendations for brain applications were recently published, such as HARNESS (HARmoNising Brain Imaging MEthodS for VaScular Contributions to Neurodegeneration; www.harness-neuroimaging.org) (Heye *et al.*, 2016; Thrippleton *et al.*, 2019). By choosing the extended Tofts model, we confirmed that *K*_trans_ evaluation was not biased by CBF, rBV or T1.

We also showed that maps for relative *K*_trans_ still enabled diagnosis of increased permeability, which could be useful in the emergency stroke context where T1 measurements may be too time-consuming.

### Importance of new findings regarding the dynamics of ischaemia–reperfusion damage

The penumbra and progression regions showed both moderately decreased ADC values and elevated *K*_trans_ values in the acute phase. In all compartments, *K*_trans_ values normalized 1 week later (Fig. 3). Whereas Zhang *et al.* (2017) found biphasic BBB leakage (as shown by MMP9 kinetics) in a similar tMCAO model in Rhesus monkey, we did not find any permeability rebound at 1 week, but we did confirm early response, assessed by AGuIX^®^ NPs permeability parameters in the acute infarct. Further studies may be necessary to explore later time points and confirm this progression. In clinical situations such as reversible vasoconstriction syndromes, extravasation of gadolinium contrast agents in the paravascular cerebrospinal-fluid space was also found to be more prevalent at earlier time points (Cho *et al.*, 2020). As demonstrated recently in a permanent occlusion rodent model, cerebrospinal fluid (CSF) influx plays an essential role in early ischaemic stroke oedema, as a consequence of vasoconstriction (Mestre *et al.*, 2020). It would be interesting to further investigate if the increased leakage after recanalization involves similar mechanisms.

### Theranostic perspectives for NPs *K*_trans_ measurement

NPs are expected to be future drug carriers across the BBB thanks to their unique transport modalities (Dong, 2018). In cancer, NPs, and especially AGuIX^®^ NPs, are able to penetrate deep into the tumour (Moura *et al.*, 2017). Small NPs can even enter cancer cells thanks to specific transport, such as micropinocytosis, as found in an earlier study with AGuIX^®^ NPs (Rima *et al.*, 2013, Kotb *et al.*, 2016*a*). Transmembrane efflux transporters such as P glycoprotein (P-gP) are also a major challenge for drug delivery, as they actively remove drugs either back to the blood or through the blood-CSF barrier to the CSF (Basseville *et al.*, 2016). NPs are thus of great interest for drug delivery, as they are able to escape this system (Dong, 2018) and may use immune cells for passage. They can also reach blood–brain interfaces through circumventricular organs such as the choroid plexus. We observed baseline *K*_trans_ values in the choroid plexus that were higher than or similar to Gd-DOTA, distinct from the proportional difference observed in brain parenchyma regions at the same time. This higher value could be due to the larger fenestration size. Alternatively, it may result from the active transport of AGuIX^®^ NPs. Moreover, the high level of immune cell trafficking in choroid plexus, which peaks during the first week after ischaemia–reperfusion, may confer a potential role for immune cells in NPs transport (Moura *et al.*, 2017; Hubert *et al.*, 2019).

### Limitations of the study

We acknowledge some limitations. First, the drug effect would need to be confirmed with a larger group of both genders. Here, males were selected because of their weight enabling the intravascular intervention (Debatisse *et al.*, 2020). Second, in large clinical trials, CT is the preferred imaging modality of the acute phase and not MRI. Moreover, a recent CT method demonstrated in patients its ability to measure lesion’s water uptake, an indicator of malignant oedema (Minnerup *et al.*, 2016; Broocks *et al.*, 2019). Yet, MRI with DCE measurements is highly relevant in a certain number of indications, for example, to expand the therapeutic window for patients with confirmed maintenance of BBB integrity. In the future, DCE MRI could also be a major surrogate biomarker to evaluate neuroprotective therapies targeting ischaemia–reperfusion damage. Finally, the AGuIX^®^ nanoparticles are not approved yet for clinical use as diagnostic agents, but the results of their first clinical trial in cancer are very promising to envisage their future clinical use (Verry *et al.*, 2020).

### Perspectives for translational stroke research in the thrombectomy era

Reproducible large animal endovascular models of stroke are challenging but are essential in translational stroke research for both pathophysiological understanding of the ischaemia–reperfusion damage and for drug testing (Herrmann *et al.*, 2019) and complied with the STAIR recommendations (Jovin *et al.*, 2016; Savitz *et al.*, 2019). As previously observed, in our model (Debatisse *et al.*, 2020), acute infarcts were generally not as massive as in human ischaemic stroke limiting the occurrence of malignant oedema, and the few cases with very large lesion died prematurely. With no massive oedema at Day 7, the non-linear registration performed well to correct for minor oedema as observed in our previous study (Debatisse *et al.*, 2020) and as also demonstrated in clinical data (Harston *et al.*, 2018). Between Day 7 and Day 30, there is substantial infarct size reduction but the tissue loss in the ipsilateral hemisphere was not large enough to disturb the non-linear registration to the template space.

Permeability measurements are the next in-line parameter for a comprehensive view at the level of the neurovascular unit. CT in emergency allows permeability evaluation predictive of later haemorrhage (Bivard *et al.*, 2020) and water transfer predictive of massive oedema (Minnerup *et al.*, 2016; Broocks *et al.*, 2019). Yet, its moderate sensitivity and the small size of standard contrast agents may limit its use to explore ischaemia–reperfusion damage. PET has an excellent sensitivity but suffers from lower spatial resolution. Hybrid PET-MRI imaging provides unique information regarding perfusion and quantitative functional measurements (Werner *et al.*, 2015, 2016). With PET-MRI, we confirmed that haemorrhagic transformation could be well assessed with both standard Gd-DOTA and AGuIX^®^ NPs (Supplementary Fig. 2).

For translational research, CT and MRI contrast agents are smaller than albumin-bound Evans dye, the gold standard in pre-clinical set-up with drug testing. Thanks to AGuIX^®^ NPs, we confirmed in NHP the decreased BBB permeability previously observed in ciclosporin A treated mice using Evans blue dye (Deng *et al.*, 2020). For further studies to repurpose ciclosporin A, it should be noticed that the single small dose injection we used may have limited the long-term benefit on the infarct size reduction that was observed in the mouse study (Deng *et al.*, 2020).

## Conclusion

This non-human primate model mimicked many features of the clinical post-thrombectomy situation, including the wide range of infarct sizes (Debatisse *et al.*, 2020). Therefore, there was only a tendency and no significant treatment effect on infarct size. However, ciclosporin A significantly limited BBB leakage in both acute and final infarct core. In a clinical study, ciclosporin A did not show the ability to diminish infarct size in the overall group of patients but did significantly reduce Day 30 final infarct in a subgroup with proximal occlusion and effective recanalization (Nighoghossian *et al.*, 2015). Recently, in a rodent model of ischaemia–reperfusion, ciclosporin A lowered post-reperfusion permeability (Deng *et al.*, 2020). Other treatments targeting barrier integrity were previously described in rodent models (Yang *et al.*, 2015; Jiang *et al.*, 2018; Li *et al.*, 2019), but this is, to our knowledge, the first report in a gyrencephalic brain. Moreover, we found a remote effect on choroid plexus permeability to NPs that was even more pronounced 1 week later, possibly linked to modulation of the inflammatory process.

In conclusion, we validated brain permeability measurements with theranostic AGuIX^®^ NPs in a wide range of values including baseline and post-stroke progression in both infarct core and remote regions. These measurements were able to monitor the effect of drug treatment, including an original and long-lasting effect of ciclosporin A on choroid plexus permeability to these small size NPs. Ultimately, these promising AGuIX^®^ nanoparticles can be considered for the transport of neuroprotective drugs, and the imaging biomarker validated in this study can readily be used for the evaluation of neuroprotective treatments.

## Supplementary material

Supplementary material is available at *Brain Communications* online.

## Supplementary Material

fcaa193_Supplementary_Data

## Abbreviations

ADC =: apparent diffusion coefficient
BBB =: blood–brain barrier
CBF =: cerebral blood flow
CsA =: ciclosporin A
DCE-MRI =: dynamic contrast-enhanced MRI
DWI =: diffusion-weighted imaging
FLAIR =: fluid-attenuated inversion recovery
HARM =: hyperintense acute reperfusion marker
* K*_*trans*_ =: volume transfer constant between plasma and extravascular extracellular space
NPs =: nanoparticles
rBV =: relative blood volume
ROI =: regions of interest
