# Supplementary material for: PET-MRI nanoparticles imaging of blood–brain barrier damage and modulation after stroke reperfusion
Source: Brain Commun. 2020 Nov 11;2(2):fcaa193. doi: 10.1093/braincomms/fcaa193 (PMC7716090; doi:10.1093/braincomms/fcaa193)
Supplement: fcaa193_Supplementary_Data [file fcaa193_supplementary_data.pdf]

SUPPLEMENTARY MATERIALS

Table S1: MRI sequences and acquisition parameters

MRI Sequence	Voxel size (mm^3)	Number of slices	Acquisition time (min)	TR/TE (ms)	FOV (mm)
**DWI (b-values: 0. 1000)**	1.3×1.3×1.3	40	04:51	11200/68	128
**FLAIR**	0.8×0.8×0.8	144	09:20	5000/419	160
**DCE-MRI AGuIX®**	2×2×2	22	10:21<br>100 frames	5.2/2.5	128
**DCE-MRI Gd-DOTA**	2×2×2	22	6 :13<br>60 frames	5.2/2.5	128
**T1 MPRAGE**	0.6×0.6×0.6	192	14:40	2650/4.21	160

DCE-MRI: dynamic contrast-enhanced MRI; DWI: diffusion-weighted imaging; FLAIR: fluid attenuated inversion recovery; FOV: field of view; MRI: magnetic resonance imaging; TE: time of echo; TR: time of repetition.

Table S2: Physiological parameters during endovascular intervention (mean ± SD) in n=16 NHP.

Parameters	Before stroke induction	During occlusion	After recanalization
**Sevoflurane in %**	1.1±0.2	1.3±0.4	1.3±0.3
**O_2 saturation in %**	96±4	96±5	94±8
**CO_2 saturation in %**	32±5	27±3	28±5
**HR in bpm**	133±15	124±14	128±18
**BP (in mmHg):**			
**Systolic**	92±6	105±13	102±15
**Mean**	65±6	76±11	72±14
**Diastolic**	39±6	47±8	44±12

BP: blood pressure; HR: heart rate.

Supplementary Figure 1

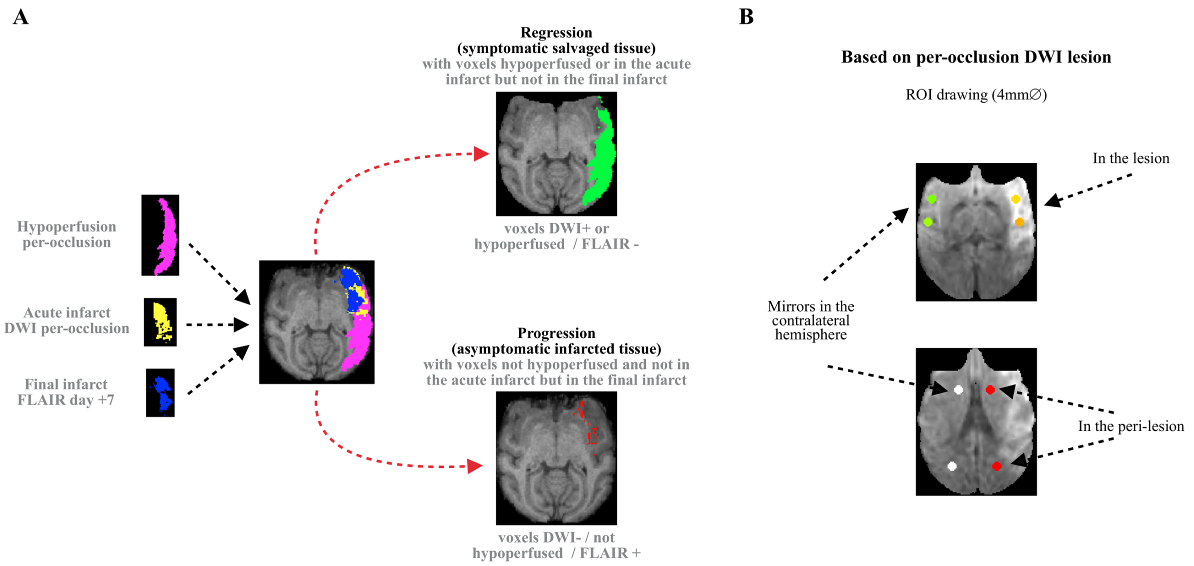

A: Definition of the two evolutive compartments with 1) regression or symptomatic salvaged tissue defined as voxels hypoperfused per-occlusion or in the acute infarct but not in the final infarct; and 2) progression or asymptomatic infarcted tissue with voxels not hypoperfused per-occlusion or in the acute infarct but in the final infarct.

B: Example of acute infarct (DWI per-occlusion) ROI definition in the lesional and peri-lesional area and corresponding contralateral ROI.

DWI: diffusion weighted imaging; ROI: region of interest.

Supplementary Figure 2

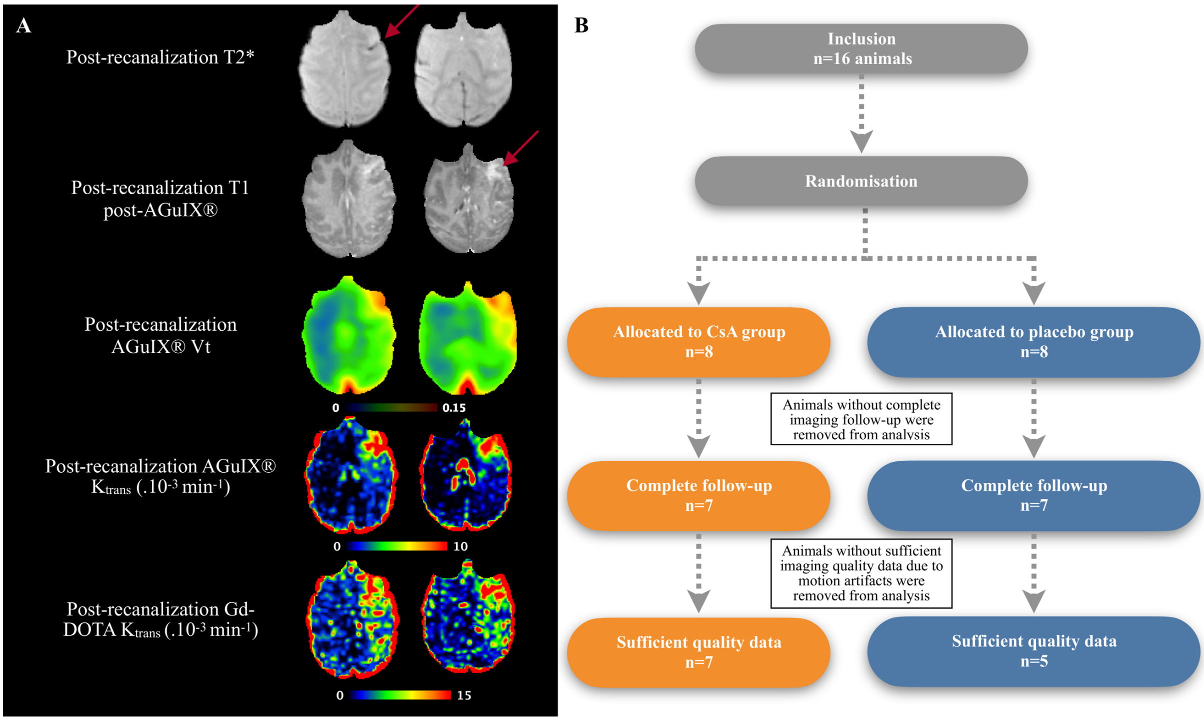

A: Illustration of one animal with hemorrhagic transformation. Two ROIs were placed in the hemorrhagic area. We found a mean K_{trans} value of 9.5 (±2) x10^{-3} min^{-1} with AGuIX® NPs and 16.6 (±5.4) x10^{-3} min^{-1} with Gd-DOTA in the hemorrhagic core.

NPs: nanoparticles; ROI: region of interest.

B: Flow chart showing animals selection for study.

Supplementary Figure 3

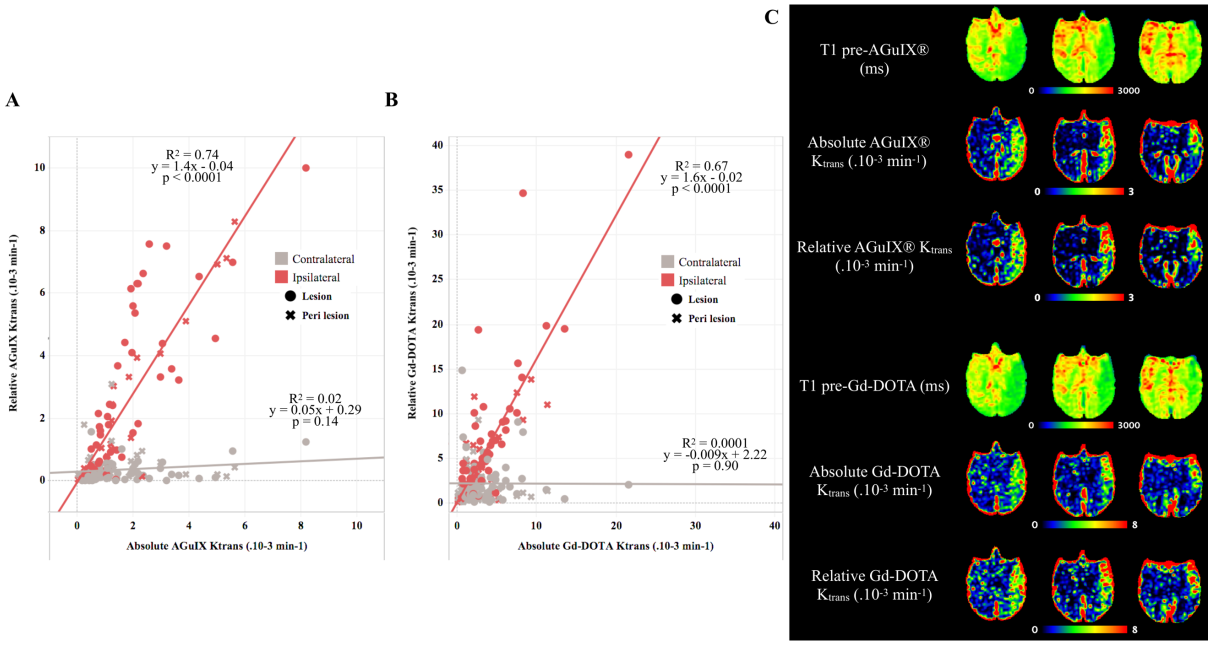

Scatter plots (**A-B**) illustrating the degree of correlation between K_{trans} and relative K_{trans} with the two contrast agents: AGuIX® NPs (**A**) in ipsilateral (red circles ; r^2=0.74; p<0.001) and contralateral (gray circles; r=0.02; p=0.14) hemispheres (n= 51 ROIs in lesions, dots; n= 45 ROIs in peri-lesion, crosses). Gd-DOTA (**B**) in ipsilateral (red circles; r^2=0.67; p<0.001) and contralateral (gray circles; r=0.001; p=0.90) hemispheres (n= 51 ROIs in lesions, dots; n= 45 ROIs in peri-lesion, crosses). Illustration (**C**) of T1 pre-contrast agents, absolute and relative K_{trans} with AGuIX® NPs and Gd-DOTA in one animal at post-recanalization.

NPs: nanoparticles; ROI: region of interest.

Supplementary Figure 4

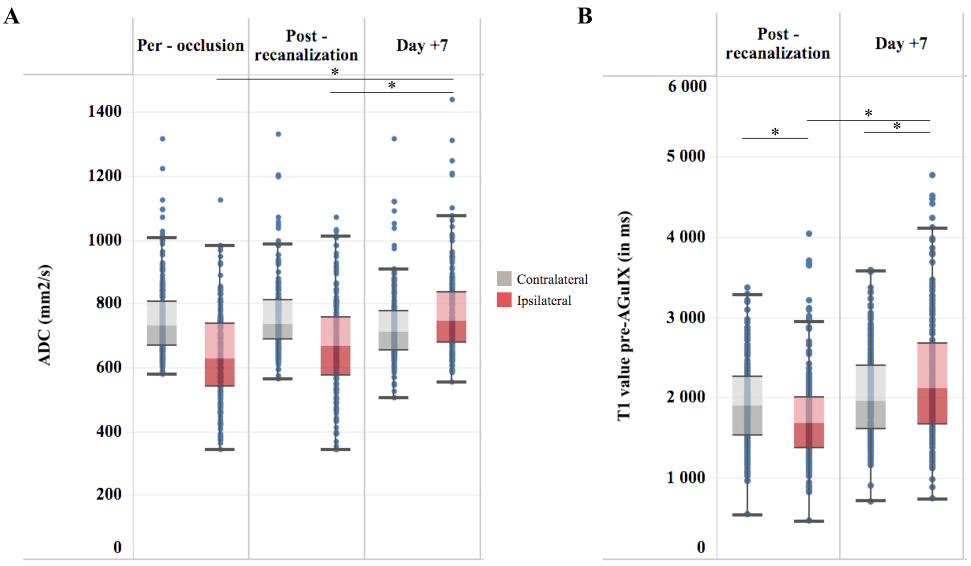

Boxplots of per-occlusion, post-recanalization, and day+7 ADC (**A**), or pre-contrast T1 (**B**) values in ROIs (n=93) in ipsilateral (red) and corresponding contralateral (gray) hemisphere.

A: * $p<0.05$, Friedman test and Dunn test for multiple comparisons

B: * $p<0.05$, paired Wilcoxon test

ADC: apparent diffusion coefficient; ROI : region of interest.

Supplementary Figure 5

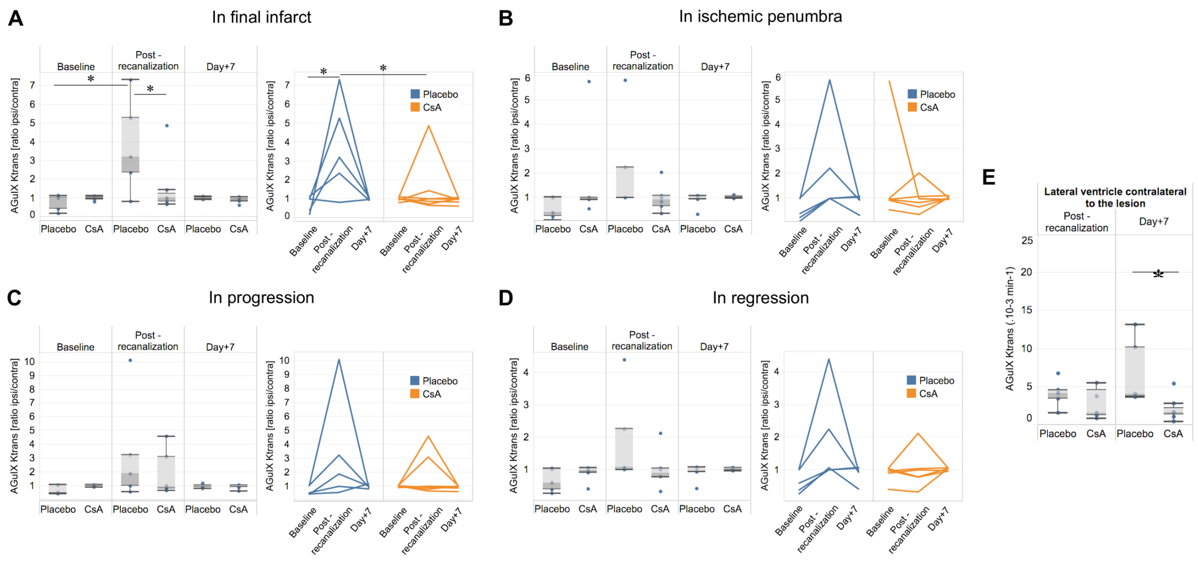

Longitudinal evolution in the final infarct (**A**), ischemic penumbra (**B**), progression (**C**) and regression regions (**D**) of normalized AGuIX® NPs K_{trans} in the two treatment groups (n=5 animals in the placebo group and n=7 animals in the CsA group) represented as boxplots (left) or individual values (right). Although not significant, the choroid plexus in the hemisphere contralateral to the lesion (**E**) showed decreased post-recanalization K_{trans} in the CsA-treated group, which persisted and became significantly lower at day 7 in the group treated with AGuIX® NPs.

A, B, C: * $p<0.05$ two-way repeated measures ANOVA followed by post-hoc Bonferroni test for multiple comparisons.

D: * $p<0.05$ non-parametric Mann-Whitney test.

NPs: nanoparticles; ANOVA: analysis of variance
